# Clinical and Three‐Dimensional Evaluation of Calcium Hydroxylapatite for Temporal Contour Restoration in a Chinese Population

**DOI:** 10.1111/jocd.71026

**Published:** 2026-07-08

**Authors:** Dong Zhu, Junsheng Chen, Wuhan Wei, Jinyi Zhou, Yunhu Ma, Xiaoyue Li, Aijun Zhang, Yanping Guo

**Affiliations:** ^1^ Department of Plastic Surgery Affiliated Hospital of Xuzhou Medical University Xuzhou Jiangsu China; ^2^ Plastic Surgery Hospital Chinese Academy of Medical Sciences and Peking Union Medical College Beijing China; ^3^ The Second Affiliated Hospital, Jiangxi Medical College Nanchang University Nanchang Jiangxi China

**Keywords:** 3D volumetric analysis, CaHA (calcium hydroxylapatite), Chinese population, facial contouring, temporal hollowing

## Abstract

**Background:**

In East Asian facial aesthetics, temporal fullness is a critical determinant of a smooth upper facial contour and a youthful appearance. Temporal hollowing disrupts the ideal oval facial silhouette and its three‐dimensional projection. While calcium hydroxylapatite (CaHA) is widely used for soft tissue augmentation, high‐level clinical evidence specifically supporting its efficacy and durability for temporal restoration in Chinese populations remains scarce.

**Objective:**

This study aimed to evaluate the efficacy, safety, and durability of a carboxymethylcellulose (CMC)‐based CaHA filler for correcting temporal hollowing in a Chinese cohort.

**Methods:**

Subjects received precise temporal injections of CaHA, exhibiting immediate structural volumetric support and potential long‐term tissue regeneration effects observed in clinical practice. Outcomes were systematically evaluated using the Temple Hollowing Scale (THS), Global Aesthetic Improvement Scale (GAIS), 3D volumetric scanning, and facial vector displacement analysis.

**Results:**

Treatment resulted in a significant increase in temporal volume, which was excellently maintained at the 12‐month follow‐up. A concomitant composite rejuvenation effect was observed, which manifested as potential tightening and lifting trends of the mid and lower face, mild eyebrow elevation, and mitigation of periorbital wrinkles. Adverse events were predominantly mild, transient, and injection‐related; for example, swelling, pain, bruising, resolving spontaneously within 1 week. No serious adverse events were reported.

**Conclusions:**

Temporal augmentation with CMC‐based CaHA is an effective, safe, and durable approach for restoring temporal contour in Chinese patients. The treatment achieves comprehensive facial aesthetic improvement, which may be associated with biomechanical contour modification and sustained structural support.

## 1 Introduction

In East Asian aesthetics, temporal fullness is highly valued, as it contributes significantly to the desirable oval‐shaped face [1, 2, 3]. Temple hollowing disrupts this ideal facial silhouette and its three‐dimensional projection. Consequently, Chinese women typically prefer a convex forehead with full temples, seeking a smooth, uninterrupted transition from the forehead to the temporal region without any concavity [4]. Precise augmentation of the superior temporal region has thus emerged as a key procedure for enhancing facial three‐dimensionality and restoring a youthful appearance [5]. An increasing number of studies are exploring the injection of dermal fillers into the temporal area to achieve this satisfactory facial contour.

**FIGURE 1:**
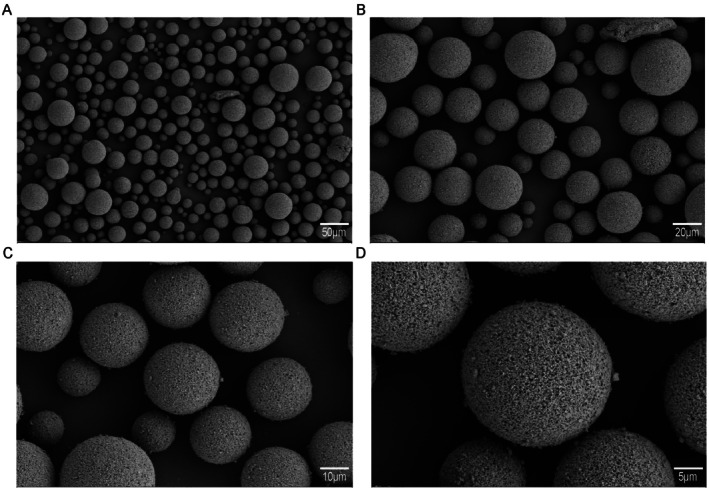
Scanning electron microscopy (SEM) images of spherical particles at different magnifications. (A) Low‐magnification view showing the size distribution and overall morphology of the spherical particles (scale bar: 50 μm, magnification: 200×). (B) Intermediate‐magnification image displaying the uniform spherical shape of the particles (scale bar: 20 μm, magnification: 500×). (C) High‐magnification view revealing the fine surface texture of the particles (scale bar: 10 μm, magnification: 1000×). (D) High‐resolution SEM image showing the porous or granular surface structure of individual spherical particles (scale bar: 5 μm, magnification: 2000×). Reproduced with permission from Shenzhen Maijie Life Science Co. Ltd. (Shenzhen, China). CaHA‐CMC, Calcium Hydroxylapatite‐Carboxymethylcellulose.

Calcium Hydroxylapatite (CaHA) is a biocompatible material with a broad clinical application history. Its first documented use was in urological conditions [6], followed by applications in treating scleroderma [7]. In recent years, with the growing demand for facial rejuvenation, CaHA has become the second most frequently administered facial injectable filler in the United States, second only to hyaluronic acid (HA) [8]. In clinical practice, CaHA microspheres can be diluted with HA or suspended within a carboxymethylcellulose (CMC) carrier gel [9]. Our study utilizes a CaHA‐based product consisting of microspheres (25–45 μm in diameter) suspended in a 70% CMC gel (Figure 1, Figure S1). This formulation provides immediate post‐procedural volume from the CMC carrier. Subsequently, CaHA microspheres may serve as a potential biostimulant that is associated with neocollagenesis in clinical observations [10]. Over 6–12 months, the newly formed collagen gradually replaces the degraded CMC, enabling long‐term maintenance of the restorative effect. Approximately 9–12 months later, the CaHA particles gradually degrade into calcium and phosphate ions, which are safely eliminated via the renal system. The processes of new collagen deposition, elastogenesis, angiogenesis, and dermal cell proliferation collectively contribute to the formation of new tissue, yielding durable aesthetic outcomes reported to last up to 18 months [11, 12].

Previous studies have demonstrated the effectiveness of CaHA for correcting volume loss in the mid‐face, nasolabial folds, hand rejuvenation, and HIV‐associated lipoatrophy [13, 14, 15]. However, there is a paucity of literature specifically investigating CaHA as a collagen‐stimulating filler for temple augmentation to correct temporal hollowing. Therefore, this study aims to evaluate the efficacy, safety, and durability of a CMC‐based CaHA filler for treating temple hollowing in a Chinese population, providing clinical data and experience for this specific aesthetic application.

## 2 Materials and Methods

### 2.1 Treatment

A total of 36 subjects assessed by the investigators as having Temple Hollowing Scale (THS) grades 2 (mild), 3 (moderate), or 4 (severe) were enrolled from the Department of Plastic Surgery, Affiliated Hospital of Xuzhou Medical University Hospital, between April 2024 and April 2025. The study protocol adhered to the principles of the Declaration of Helsinki and was approved by the hospital's Ethics Committee (Approval No. XYFY2023‐QL176). Written informed consent was obtained from all participants. Eligible subjects were randomized in a 2:1 ratio to either the experimental (CaHA treatment) group or the control group, using a central interactive web response system (IWRS). Subjects in the control group received no treatment, while those in the experimental group received injections of the CaHA dermal filler (containing 1.0 mL CaHA per milliliter).

The injection procedure followed a standardized, dual‐plane approach using both needles and cannulas to address different anatomical requirements. Initially, a 27G × 13 mm needle was utilized for deep supraperiosteal injections in the upper temporal region, specifically targeting the temporal–forehead junction. To ensure structural support and safety, small boluses (typically 0.1–0.2 mL per point, 2–3 points per side) were deposited perpendicularly onto the periosteum after confirming the absence of blood aspiration. Subsequently, a 23G × 50 mm blunt cannula was introduced via a zygomatic arch entry point to treat the middle‐to‐lower temporal region. Following the injections, gentle massage was performed to ensure even distribution of the filler. Standardized digital photographs were taken at baseline, immediately after treatment, and at 1, 6, and 12 months post‐treatment. A touch‐up injection was permitted at the 1‐month follow‐up visit if the desired outcome was not achieved and subject consent was obtained.

### 2.2 Efficacy Evaluation

All subjects underwent THS assessments at baseline and each follow‐up visit. They were also guided to complete the Global Aesthetic Improvement Scale (GAIS) at these time points. Three‐dimensional images of the entire face were captured immediately post‐injection and at all subsequent follow‐ups using the Vectra H2 Mirror system. The Geomagic software (Morrisville, USA) was utilized to calculate volumetric changes across different visits. All follow‐up scans were automatically co‐registered with their respective baseline scans to compute surface projections and vector skin displacement differences for each investigated facial region. Procedure outcomes were analyzed both locally (i.e., within individual injection areas) and regionally (i.e., across multiple injected facial areas).

The primary efficacy endpoint was the THS improvement rate at 12 months compared to baseline. As each subject received bilateral (left or right) injections, the worst result from either side was used for the final efficacy evaluation of this primary endpoint. The THS improvement rate was assessed by both the investigators and an Independent Review Committee (IRC), composed of qualified and authorized professionals from the Affiliated Hospital of Xuzhou Medical University, based on the captured photographs. The THS improvement rate at Month 12 was calculated as follows: (Number of effective cases/Total number of cases in the group) × 100%.

### 2.3 Safety Evaluation

Subjects rated the pain experienced during the procedure using an 11‐point numerical rating scale (0 = no pain to 10 = worst pain imaginable). All adverse events were self‐reported and recorded daily by patients for 14 days following the injection. The severity (graded as mild, moderate, or severe) and duration (in days) of injection site reactions were documented.

### 2.4 Statistical Analysis

Statistical analyses were performed using SPSS 20 (Chicago, USA), and graphs were generated with GraphPad Prism 9 (California, USA). The normality of the data distribution was assessed using the Shapiro–Wilk test. Comparisons were made using Student's *t*‐test, paired‐sample *t*‐test, or one‐way analysis of variance (ANOVA), as appropriate. A two‐sided *p*‐value of < 0.05 was considered statistically significant.

## 3 Results

### 3.1 Subject Characteristics

This study enrolled 36 Chinese female subjects with Temple Hollowing Scale (THS) grades 2–4. Participants were randomized into a control group (*n* = 12) and a CaHA treatment group (*n* = 24). All subjects completed the study and follow‐up. The mean age was 44.33 ± 9.04 years in the control group and 47.83 ± 6.91 years in the CaHA group, with no statistically significant difference between the groups (*p* = 0.36). The baseline THS scores were 2.91 ± 0.79 for the control group and 3.00 ± 0.82 for the CaHA group, indicating no significant difference (*p* = 0.55). Detailed baseline characteristics are presented in Table 1.

**TABLE 1:** The baseline demographics of all subjects.

Characteristics	Control (*n* = 12)	CaHA (*n* = 24)	*p*
Gender			
Female, *n* (%)	12 (100)	24 (100)	—
Age	44.33 ± 9.04	47.83 ± 6.91	0.36
Temple Hollowing Scale	2.91 ± 0.79	3.00 ± 0.82	0.55
Scale = 2, *n* = (%)	4 (33)	7 (29)	
Scale = 3, *n* = (%)	5 (42)	8 (33)	
Scale = 4, *n* = (%)	3 (25)	7 (29)	

### 3.2 Injected Volumes of CaHA

Among the 24 subjects in the CaHA group, 4 (16.7%) received a single injection session, while 20 (83.3%) received two sessions. This high touch‐up rate reflects our treat‐to‐target protocol rather than a single‐session regimen. To evaluate the clinical driver for touch‐up interventions, we analyzed the baseline severity between these two subsets. Subjects who required a secondary touch‐up session presented with a significantly higher baseline severity of temporal hollowing compared to those who achieved optimal correction in a single session (mean baseline THS score: 2.90 ± 0.72 vs. 2.00 ± 0.00, respectively). The mean total injection volume of CaHA was 3.60 ± 0.90 mL. During the first injection session, the maximum and minimum unilateral volumes were 2.0 and 1.4 mL, respectively. For subsequent touch‐up injections, the maximum and minimum unilateral volumes were 2.0 mL and 0.2 mL, respectively. The mean injected volume was 1.88 ± 0.21 mL for the right temple and 1.90 ± 0.20 mL for the left temple (*p* = 0.78). Detailed injection data are summarized in Table 2.

**TABLE 2:** Volumes of CaHA injected.

	Single‐injection	Additional‐injection	Total
Volume injected Mean ± SD			
Right	1.88 ± 0.21	1.29 ± 0.51	3.60 ± 0.90
Left	1.90 ± 0.20	1.41 ± 0.39	
Minimum			
Right	1.4	0.2	3.0
Left	1.4	0.8	1.0
Maximum			
Right	2.0	2.0	4.0
Left	2.0	2.0	4.0

### 3.3 Improvement in Temple Hollowing and Global Aesthetic Appearance

**FIGURE 2:**
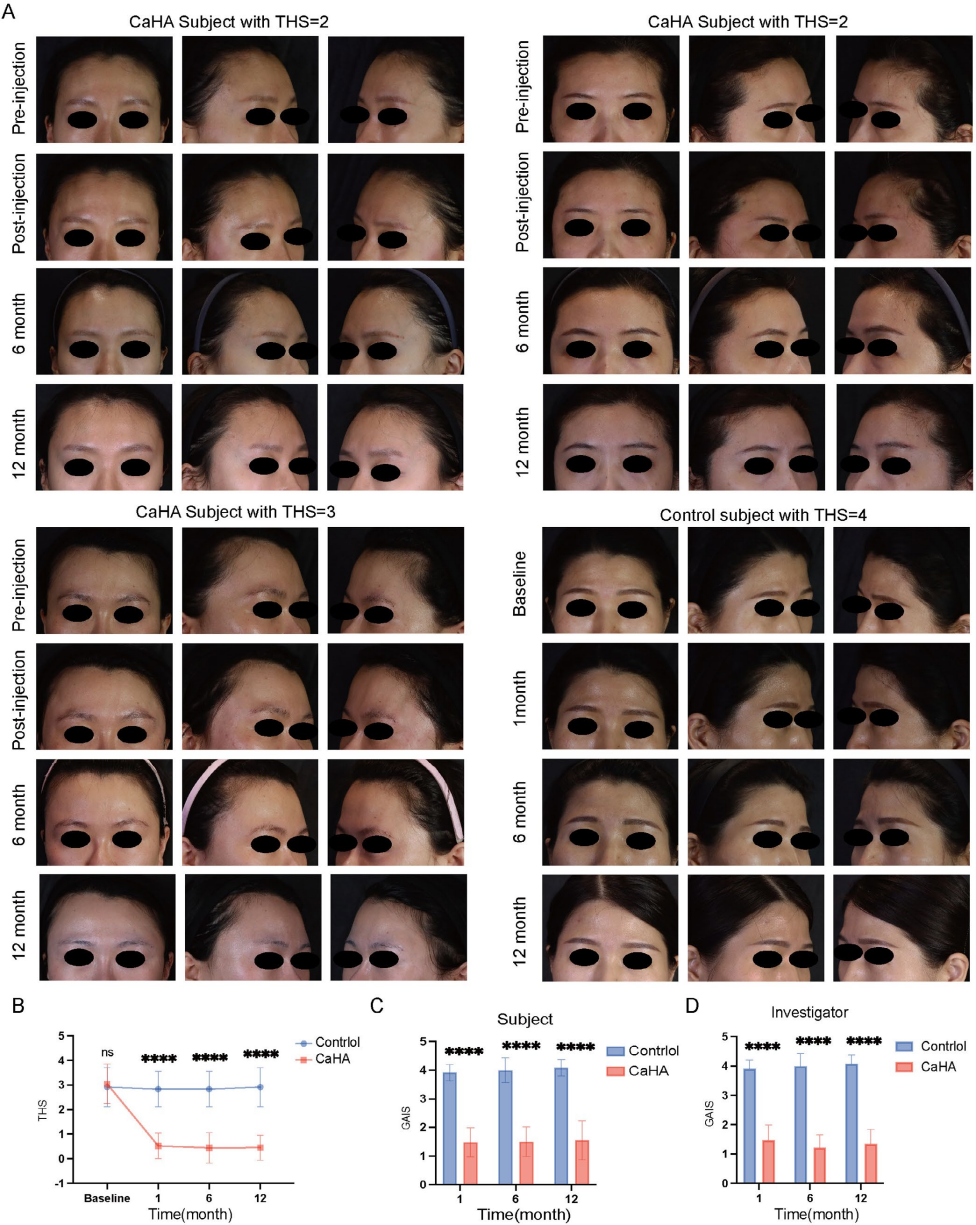
Temple Hollowing Scales (THS) and Global Aesthetic Improvement Scales (GAIS) in the CaHA group and control group. (A) Representative photographs of subjects with THS = 2, 3, 4 in the CaHA group and subjects with THS = 4 in control groups. (B) Comparison of investigator‐assessed between the CaHA group and control group. (C) Comparison of investigator‐assessed GAIS and subject‐assessed GAIS between CaHA group and control group. ****, *p* < 0.0001. ns, not significance.

Standardized photographs were taken before and after treatment during all study visits. Representative cases from three subjects are shown in Figure 2A. Compared to the control group, the CaHA treatment group demonstrated significant improvement in THS scores throughout the follow‐up period (Figure 2B). Both subject‐assessed and investigator‐assessed Global Aesthetic Improvement Scale (GAIS) scores showed significant improvement at the 1‐, 6‐, and 12‐month follow‐ups compared to the control group (Figure 2C,D).

### 3.4 Global Facial Tightening Effect Following Temporal CaHA Injection

**FIGURE 3:**
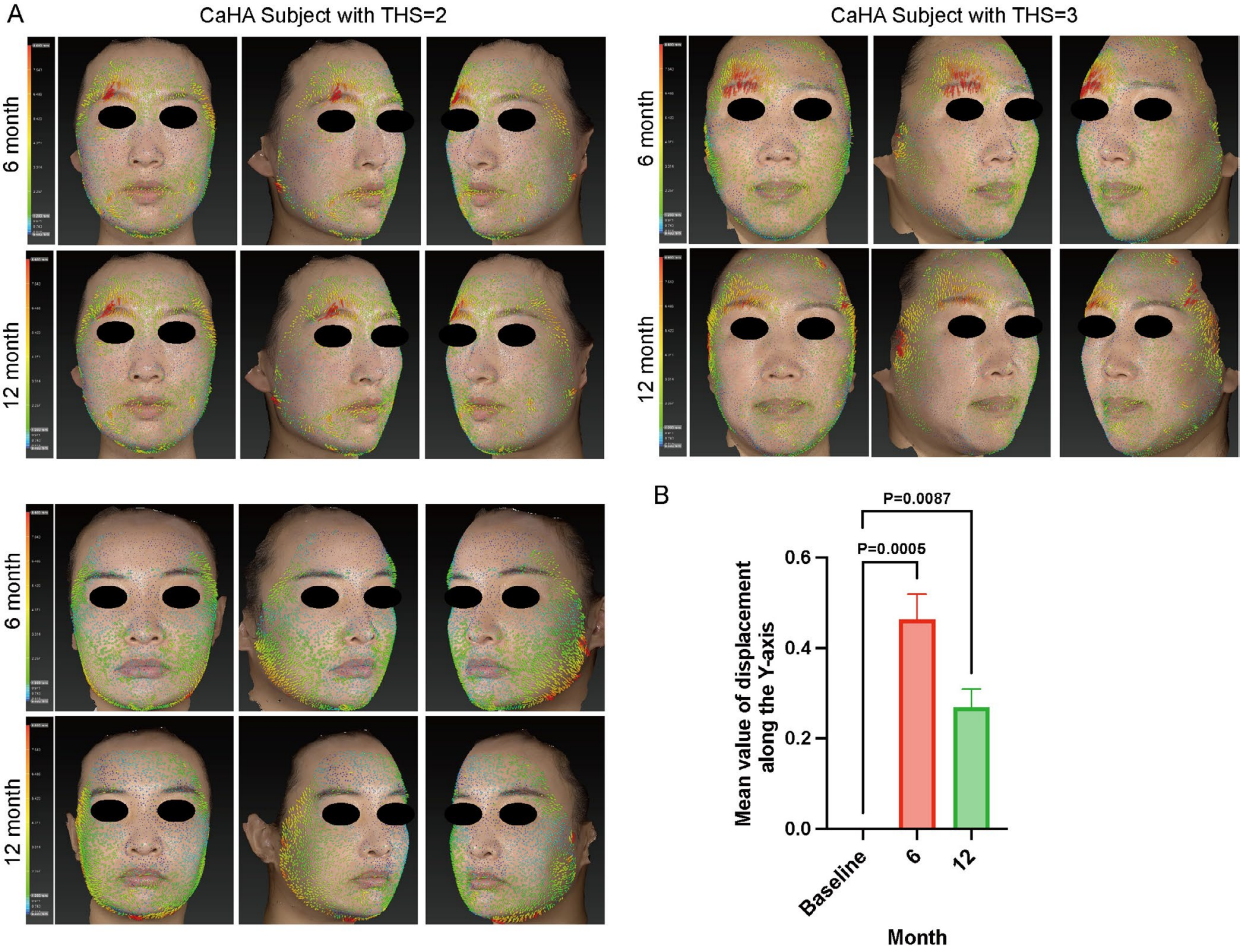
Three‐dimensional photographs of three treated female patients, with superimposed colored skin vector displacements to illustrate skin movement. Note that the vectors point toward the temples, with eyebrow elevation observed. The length of the vectors corresponds to the amount of the skin displacement that has taken place in the direction of two‐dimensional X and Y axes; the color of the vectors corresponds to the local volume increase (red) or decrease in volume (blue), *n* = 3. **, *p* < 0.01, ***, *p* < 0.001.

Building upon previous research indicating that temporal filler injection can improve temple hollowing and eyebrow position [16]. we further analyzed three‐dimensional photographs. This analysis revealed an outward and upward vector of skin displacement post‐injection. The clinical correlates of this biomechanical effect included eyebrow elevation, reduction in crow's feet, and a notable tightening and lifting of the mid and lower face, accompanied by an apparent reduction in mid‐facial volume (Figure 3A).

### 3.5 Volume Retention Analyzed by Three‐Dimensional Imaging

**FIGURE 4:**
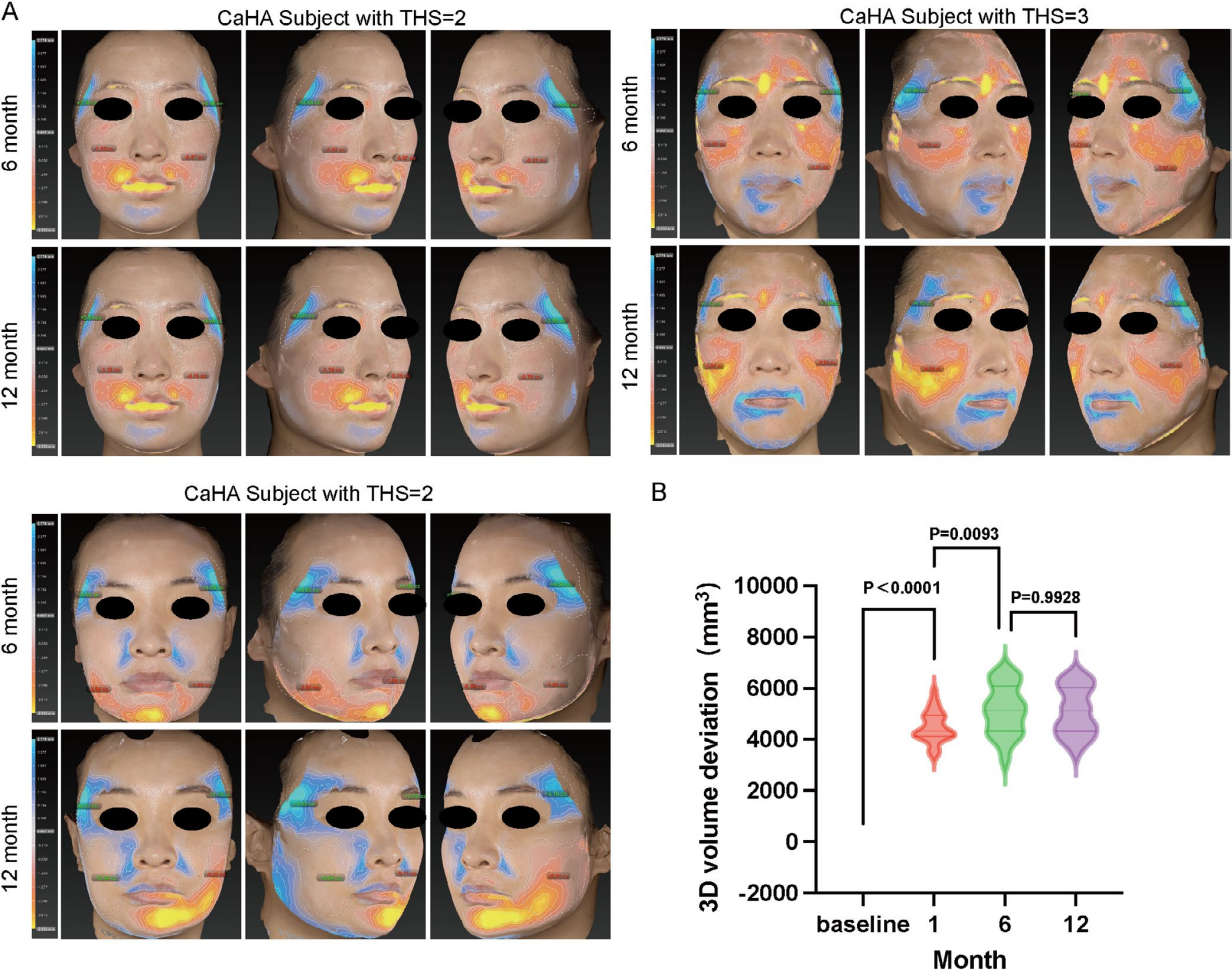
Temporal volume changes in the CaHA group were assessed using a three‐dimensional scanner. (A) Representative results from a subject in the CaHA group. Blue and orange colors indicate volume increase and decrease, respectively, in the simulated overlay comparing pre‐ and post‐injection images processed with Geomagic software. (B) Comparison of three‐dimensional volume changes at 1, 6, and 12 months post‐injection. Statistical significance was determined using one‐way repeated measures ANOVA followed by Šídák's post hoc test for multiple comparisons.

Volumetric changes were assessed using 3D imaging. The mean actual injected volume of CaHA was 3600.00 ± 900.00 mm^3^. Excellent volume retention was observed in the temporal region during the 12‐month follow‐up period. Statistical analysis of 3D volume deviation showed that while temporal volume significantly increased from baseline to Month 1 (*p* < 0.0001), there was no significant difference between Month 6 and 12 (*p* = 0.9928), indicating stable long‐term support (Figure 4A,B). Notably, the increase in temporal volume was accompanied by a significant reduction in volume in the zygomatic (mid‐face) and perioral areas, resulting in a flatter and more refined mid‐facial contour. These findings suggest that temporal augmentation with CaHA can induce a repositioning effect on the mid and lower facial tissues, leading to an overall tightening and refining of the facial contour.

### 3.6 Safety and Adverse Events

Common adverse events were localized reactions, including swelling, erythema, and pain. No severe adverse reactions, granulomas, or foreign body reactions were reported within the 6‐month safety follow‐up period. The specific incidence of treatment‐related adverse events is summarized in Table 3. Most adverse events were mild and transient, resolving within a few days, and did not lead to study withdrawal. No serious adverse events, such as vascular thrombosis, skin necrosis, or blindness, occurred during the study.

**TABLE 3:** Treatment‐related AEs.

Treatment‐related AEs	CaHA (*n*, %)
Pain	2 (8)
Tenderness	3 (13)
Topical swelling	6 (25)
Intermittent periorbital edema	2 (8)
Itchiness	1 (4)
Redness	1 (4)

## 4 Discussion

In East Asian aesthetics, the oval face shape is highly prized, and concavities in the forehead and temples are perceived as disrupting this ideal facial silhouette, its three‐dimensional projection, and contributing to an aged and unhealthy appearance [3]. The desire for full temples and a convex forehead is also closely linked to cultural beliefs in facial physiognomy, with approximately 63% of the general population and 86.3% of aesthetic practitioners in relevant regions expressing belief in its principles [4, 17, 18]. The temporal region serves as a critical transition zone between the upper and mid‐face, and its contour is fundamental to overall facial harmony. Temporal hollowing is recognized as an early sign of facial aging [19, 20]. In Asian populations, characterized by flatter facial bone structure and thinner subcutaneous fat layers, temporal hollowing is not only more prevalent but also more likely to create visual imbalances, such as accentuated zygomatic prominence and disrupted facial proportions, thereby diminishing facial three‐dimensionality and youthfulness.

Autologous fat grafting was an early mainstay for temporal augmentation but faced challenges like unpredictable resorption and the inherent invasiveness of a surgical procedure [21]. Subsequently, hyaluronic acid (HA) fillers gained popularity due to their minimal downtime and immediate results. Our previous research reported on a novel amino acid crosslinked hyaluronic acid (ACHA) for effectively treating temple hollowing [22], While effective, fillers primarily based on HA or similar hydrogels may have limitations in providing robust structural support and inducing profound, long‐term neocollagenesis necessary for ultra‐long maintenance (> 18 months). Furthermore, while studies have reported mid and lower facial lifting effects achieved by injecting fillers in the lateral temporal region to support the superficial temporal fascia, the efficacy of this lifting is highly dependent on the patient's skin laxity. In cases of significant laxity, temporal augmentation may improve volume but fail to produce a meaningful lift [23]. Consequently, for patients presenting with both skin laxity and temporal hollowing, effective treatment requires a strategy that combines skin tightening with volume restoration.

In recent years, the concept of “long‐acting regenerative filling” has gained momentum, shifting research interest toward biostimulatory materials [24]. The CaHA‐CMC composite used in this study features smooth, uniform spherical microspheres predominantly ranging from 20 to 45 μm in diameter [25]. Evidence indicates that CaHA microspheres not only provide immediate structural support but also promote collagen production, enabling longer‐lasting results, stronger deep tissue support, a lower risk of postoperative edema, and superior biostimulatory capacity [26].

Since its initial reported use in urology, CaHA has expanded its applications to areas like scleroderma treatment, leveraging its excellent biocompatibility and tissue‐inducing properties [27]. Its core value lies in the dual‐mode effect of its microspheres, offering both physical scaffolding and collagen regeneration. Radiesse, a sterile, latex‐free, non‐pyrogenic, semi‐solid, cohesive, subdermal injectable implant whose principal component is synthetic CaHA, received FDA approval in 2006 for the correction of moderate to severe facial wrinkles and folds, as well as for HIV‐associated lipoatrophy [11, 28]. As the calcium‐based microspheres share a chemical composition with the mineral phase of human bone and teeth, CaHA is highly biocompatible and typically does not require pre‐treatment sensitivity testing. Furthermore, it does not obscure computed tomography (CT) imaging, posing no threat of confounding radiological findings [29]. Although some concerns regarding the safety of CaHA have been raised, numerous studies have substantiated its safety profile as a facial filler [25, 26, 27].

In the present study, we utilized an undiluted CaHA microsphere/CMC gel injectable for temporal augmentation. This high G' material is well‐suited for providing the necessary support and volume correction in the temples [30, 31, 32, 33]. Our volumetric analysis using the Vectra 3D imaging system revealed a noticeable volume reduction at 1 month post‐injection compared to the immediate post‐procedure state, which we attribute to the metabolic clearance of the CMC gel carrier. This transient phenomenon, termed the “settling effect” in some clinical contexts, has been documented in other studies [34]. However, at the 6‐ and 12‐month follow‐ups, temporal volume demonstrated a significant increase, indicating that the potential collagen neogenesis associated with CaHA microspheres may compensate for the initial volume loss from the CMC.

We also observed that patients receiving only a single treatment session exhibited lower volume retention rates compared to those who received a supplementary injection, suggesting that touch‐up treatments may be beneficial for biomechanical lifting effect and sustaining the aesthetic outcome. A noteworthy finding was the concurrent reduction in volume observed in the mid‐face and perioral regions alongside temporal augmentation. This finding suggests that temporal CaHA injection may contribute to observed mid‐ and lower‐facial tissue repositioning and tightening trends. Vector displacement analysis corroborated this, demonstrating eyebrow elevation and improved skin tightness in the mid and lower face at 6 and 12 months.

Regarding safety, the adverse events (AEs) observed in this study were predominantly mild and localized, including swelling, pain, bruising, and induration at the injection site. The majority of these AEs resolved spontaneously within 5–7 days, with none persisting beyond 2 weeks. During long‐term follow‐up, two subjects reported intermittent periorbital edema starting approximately 2 months post‐injection, the severity of which fluctuated with their general physical condition. Both subjects had a documented history of allergies (to milk and eggs, respectively). The edema responded well to oral prednisone acetate and resolved completely by 6 months post‐injection, with no recurrence thereafter. This late‐onset intermittent edema represents a relatively rare delayed adverse event, which may be related to individual hypersensitivity and low‐grade local inflammation. The favorable response to oral corticosteroids suggests a potential immune‐mediated mechanism. Patients with preexisting allergic histories may require more careful counseling and prolonged observation to identify and manage such delayed reactions promptly.

In conclusion, this study demonstrates that CaHA is a safe and effective treatment for temporal hollowing in a Chinese cohort, as validated by improvements in THS and GAIS scores, 3D volumetric analysis, and facial vector assessment. CaHA, with its established long‐term efficacy and safety profile in Western populations sustained for up to 4 years [35, 36], shows great promise for application in Asian facial aesthetics. Nonetheless, our study has several limitations. The relatively small sample size and the lack of long‐term follow‐up data beyond 24 months necessitate further investigation with larger cohorts and extended observation periods to confirm the long‐term safety and durability of CaHA in the temporal region. Secondly, refining individualized dosing protocols based on the severity of hollowing (mild, moderate, severe) is crucial for optimizing treatment precision. Finally, numerous studies suggest that diluting CaHA to different concentrations allows for tailored treatments targeting different tissue layers and facial areas, facilitating comprehensive facial rejuvenation [30, 33, 37]. Future research will focus on more precisely defining the role and protocols for CaHA in the broader context of facial rejuvenation for Asian populations.

## 5 Conclusion

CaHA proves to be an effective and well‐tolerated dermal filler for temporal augmentation, demonstrating significant cosmetic improvement across mild, moderate, and severe grades of temporal hollowing in Chinese patients. Beyond restoring temporal volume, the treatment elicits notable ancillary benefits—including midfacial tightening, contour refinement, and lower facial lifting—particularly beneficial for individuals presenting with concomitant midfacial fullness and oral commissure laxity. With a favorable safety profile, CaHA‐based temporal rejuvenation represents a compelling nonsurgical option in the armamentarium of facial aesthetic restoration.

## Author Contributions

**Dong Zhu:** conceptualization, literature searches, writing – original draft preparation. **Junsheng Chen** and **Wuhan Wei:** investigation, visualization. **Jinyi Zhou:** data analysis. **Yunhu Ma** and **Xiaoyue Li:** literature searches. **Aijun Zhang:** visualization and writing‐reviewing and editing. **Yanping Guo:** conceptualization, supervision, writing – reviewing, and editing.

## Funding

The authors have nothing to report.

## Ethics Statement

This study was approved by the Ethics Committee of the Affiliated Hospital of Xuzhou Medical University (Approval No. XYFY2023‐QL176). All procedures involved in this study adhere to the principles of the Declaration of Helsinki. Written informed consent was obtained from all participants before they took part in the study.

## Consent

Informed consent was obtained from all individual participants included in the study; participants signed informed consent regarding publishing their data.

## Conflicts of Interest

The authors disclose that SEM imaging support was provided by Shenzhen Maijie Life Science (the manufacturer of the CaHA product). The authors declare no conflicts of interest.

## Supporting information


**Figure S1:** Scanning electron microscopy (SEM) images of spherical particles at specific magnifications. (A) High‐magnification view showing the size distribution and overall morphology of the spherical particles (scale bar: 2 μm, magnification: 5000×). CaHA‐CMC, Calcium Hydroxylapatite‐Carboxymethylcellulose.

## Data Availability

The data that support the findings of this study are available from the corresponding author upon reasonable request.
