# Supplementary material for: Clinical and Three‐Dimensional Evaluation of Calcium Hydroxylapatite for Temporal Contour Restoration in a Chinese Population
Source: J Cosmet Dermatol. 2026 Jul 8;25(7):e71026. doi: 10.1111/jocd.71026 (PMC13346515; doi:10.1111/jocd.71026)
Supplement: Supplementary file 1 — Figure S1: Scanning electron microscopy (SEM) images of spherical particles at specific magnifications. (A) High‐magnification view showing the size distribution and overall morphology of the spherical particles (scale bar: 2 μm, magnification: 5000×). CaHA‐CMC, Calcium Hydroxylapatite‐Carboxymethylcellulose. [file JOCD-25-e71026-s001.docx]

Supplementary Figure 1





Supplementary Figure 1 Scanning electron microscopy (SEM) images of spherical particles at specific magnifications. (A) High-magnification view showing the size distribution and overall morphology of the spherical particles (scale bar: 2 μm, magnification: 5000×). CaHA-CMC, Calcium Hydroxylapatite-Carboxymethylcellulose.
